# Editorial: Iron metabolism and biochemical cycling mediated by microorganisms

**DOI:** 10.3389/fmicb.2024.1389229

**Published:** 2024-03-05

**Authors:** Lei Yan, Sujun Li, Dong Lu

**Affiliations:** ^1^Heilongjiang Provincial Key Laboratory of Environmental Microbiology and Recycling of Argo-Waste in Cold Region, College of Life Science and Biotechnology, Heilongjiang Bayi Agricultural University, Daqing, China; ^2^School of Informatics, Computing and Engineering, Indiana University, Bloomington, IN, United States; ^3^Institute of Modern Physics, Chinese Academy of Sciences (CAS), Lanzhou, China

**Keywords:** iron metabolism, iron-reducing bacterium, archaeal methanogen, mechanism, fungi

Biogeochemical cycling of iron is crucial to many environmental processes for a very long period and takes place across a wide range of subsurface and near-surface environments, potentially encompassing millimeter-to-decametre (or more) spatial scales. Iron exists in two main redox states in nature: ferric iron, which is poorly soluble at circumneutral pH, and ferrous iron, which is generally more soluble and hence more accessible for biological processes. The redox transition between the Fe(II) and Fe(III) valence states forms a complex network of biochemical interactions, including a tight interplay of biotic and abiotic reactions, and has a fundamental role in environmental biogeochemistry.

Redox reactions involving iron can occur abiotically, as well as biotically, and it is only since the late 20^th^ century that the role of microbes has been firmly established. In recent years, many microorganisms that obtain energy from iron redox reactions have been discovered, and our understanding of their physiology, ecology, and environmental importance is expanding rapidly. This Research Topic aims to provide an overview of new insights into iron metabolism and biogeochemical cycling. The goal is to expand knowledge of iron oxidation and reduction processes and the biochemical mechanisms associated electron transport. In this topic, we have gathered contributions from scientists working in diverse disciplines with common interests in iron cycling at the process and gene levels from the archaea, bacteria, and fungi. We hope to consolidate various research strands in one publication to enhance the overall comprehension of the iron cycle and perhaps elicit new insight into the interconnections within the cycle. We were lucky to have assembled a diverse and talented team of writers to offer six original research articles.

The research article of Sklute et al. focused on the biogenic mineral products formed by the thermophilic iron-reducing bacterium *Desulfovulcanus ferrireducens* when cultured separately on ferrihydrite, akaganeite, and lepidocrocite. Mineral spectroscopy, X-ray diffraction (XRD), and electron microscopy were coupled to provide complementary analyses of the minerals in bioreduced samples following growth and in uninoculated controls. The study demonstrated that thermophilic bacteria transform different types of Fe(III) (oxyhydr)oxide minerals for growth with varying mineral products. The results help determine the mechanism used for iron reduction by *D. ferrireducens* and the potential influence of temperature and cell type (i.e., thermophilic bacterium vs. hyperthermophilic archaeon) on iron reduction. These findings also revealed that understanding the geochemical factors influencing mineral transformations and the interaction mechanism between the analytical technique and the sample is crucial for accurately interpreting potential biosignatures in laboratory bioreduction experiments.

As a model dissimilatory iron-reducing bacterium, *Shewanella putrefaciens* can grow in aerobic and anaerobic environments. The influences of temperature on *Shewanella putrefaciens* CN32 are metabolism dependent. The metabolic performance of microbes ultimately impacts geochemical conditions, which are determined mainly by the allocation of energy between growth-associated costs and non-growth-associated maintenance. Wray and Gorman-Lewis examined the bioenergetics of O_2_ and Fe(III) reduction coupled to lactate oxidation in *Shewanella putrefaciens* CN32 and presented the first quantitative comparison of aerobic and anaerobic growth. The findings improved our insights into the thermodynamic constraints of iron-reducing bacterium *Shewanella putrefaciens*.

Fe(III)-reduction research has mainly focused on bacteria rather than fungal communities. Li et al. examined the potential and diversity of iron(III)-reducing fungi by adding antibiotics to inhibit bacterial activity and ITS sequencing and genome annotation, respectively, in paddy soil. The interaction between Fe(III)-reducing fungi in response to different organic substrates in paddy soil was explored. This study offers insight into the contribution of fungi in directly driving carbon and iron biogeochemical cycling. It provides a new perspective for further understanding of carbon and iron cycling in environments.

Furthermore, the reactivation and reduction of low reactive iron oxides in deep methanogenic sediments attracted the attention of Eliani-Russak et al., specifically iron-methane couplings and microbial players, with their impacts on the carbon, iron, and other cycles. They studied the potential of the archaeal methanogen *Methanosarcina barkeri* to reduce iron in close to natural conditions. The result showed the critical role of methanophenazines in the competitive edge of methanogens over Fe(III)-reducing bacteria in reducing the iron in deep methanogenic sediments, providing new insights into the coupling of microbial iron reduction and the important greenhouse gas methane.

Iron absorption and transportation are both necessary for the survival of most organisms. Yang et al. revealed the specificity and mechanism of TonB-dependent ferric catecholate uptake by Fiu, a presumed transporter of monomeric ferric catecholates, by introducing Cys residues in its surface loops and modifying them with fluorescein maleimide (FM). The study explained the pathways of ferric catecholate uptake in *E. coli* and *A. baumannii*, and provided better comprehension on the general mechanism of metal transport through TonB-dependent LGP.

Grubwieser et al. presented the latest development in manipulating the macrophage metabolism of *Klebsiella pneumoniae* in their research. They analyzed host- and pathogen-specific genes and protein expressions of key iron metabolism molecules. The results indicated that KP can reprogram macrophage iron metabolism to acquire sufficient amounts of this essential nutrient and aid intracellular persistence. Insights into immune metabolism and its effects on this host-pathogen interaction would aid the development of novel antimicrobial therapies.

In conclusion, the Research Topic presented an overview of research on iron metabolism and biogeochemical cycling from a range of perspectives, indicating the breadth of work that has been done and providing insight into the many exciting avenues of research.

## Author contributions

LY: Writing—original draft, Writing—review & editing. SL: Writing—review & editing. DL: Writing—review & editing.

